# Fundamental Characteristics of Bat Interferon Systems

**DOI:** 10.3389/fcimb.2020.527921

**Published:** 2020-12-11

**Authors:** Emily Clayton, Muhammad Munir

**Affiliations:** Department of Biomedical and Life Sciences, Lancaster University, Lancaster, United Kingdom

**Keywords:** bats, innate immunity, interferons, host-pathogen interaction, virus transmission

## Abstract

Interferons are an essential component of the innate arm of the immune system and are arguably one of the most important lines of defence against viruses. The human IFN system and its functionality has already been largely characterized and studied in detail. However, the IFN systems of bats have only been marginally examined to date up until the recent developments of the Bat1k project which have now opened new opportunities in research by identifying six new bat genomes to possess novel genes that are likely associated with viral tolerance exhibited in bats. Interestingly, bats have been hypothesized to possess the ability to establish a host-virus relationship where despite being infected, they exhibit limited signs of disease and still retain the ability to transmit the disease into other susceptible hosts. Bats are one of the most abundant and widespread vertebrates on the planet and host many zoonotic viruses that are highly pathogenic to humans. Several genomics, immunological, and biological features are thought to underlie novel antiviral mechanisms of bats. This review aims to explore the bat IFN system and developments in its diverse IFN features, focusing mainly on the model species, the Australian black flying fox (*Pteropus alecto*), while also highlighting bat innate immunity as an exciting and fruitful area of research to understand their ability to control viral-mediated pathogenesis.

## An Introduction to the Interferon System

Interferons (IFNs) are a group of secreted cytokines that can induce an antiviral state of the host and primarily are responsible in inhibiting viral replication. They comprise part of the innate immune response and are one of the first and arguably most important lines of defence produced against viral infection, intended to limit the spread of a virus upon early infection (Weber et al., 2004). IFNs were first discovered in 1957 by Isaacs and Lindenmann (1987) who have identified interference of viral host antagonization in chick embryos. Since then, countless studies have been conducted in investigating the functional dynamics of IFNs in mammals. Three types of IFN exist in humans (type I, II, and III), which are categorized according to their amino acid sequences and their cognate receptor complex (Baker and Zhou, 2015). Type I IFNs consist of several genes including IFNα and IFNβ, which are both induced directly in response to viral infection, alongside IFNδ, IFNκ, IFNϵ, and IFNω, which all play less well-defined roles (Randall and Goodbourn, 2008). In the type II IFN group, there is only one single IFN called IFNϒ, which is secreted by T cells and natural killer (NK) cells of the immune system and is hence more associated with cell mediated immunity than innate (Randall and Goodbourn, 2008). Type III IFNs have been discovered to consist of three main members; IFNλ1, IFNλ2, and IFNλ3 which are often referred to as interleukin (IL-)29, IL-28A, and IL-28B, respectively, in addition to the recent identification of IFNλ4, which is said to resemble IFNλ3 (Uze and Monneron, 2007). Both type I and type III IFNs are activated through the same signaling pathway and are secreted by viral-infected cells to elicit an antiviral state in infected and neighboring cells and work as part of the innate immune response (Onoguchi et al., 2007). IFNs interact with specific cellular receptors on cells, which activate signal transduction pathways that ultimately lead to the transcription of antiviral and immune modulatory genes, also referred to as IFN-stimulated genes (ISGs) (Le Page et al., 2000). A subset of ISGs can be directly induced by viral infection without the aid of IFNs and provide additional protection to infected cells.

## Interferon Induction

The IFN induction and signaling processes in response to a viral infection in humans have been discussed extensively elsewhere (Randall and Goodbourn, 2008). Briefly, IFN induction is stimulated following the recognition of pathogen-associated molecular patterns (PAMPS) which are molecular structures absent in uninfected cells, essential for the survival of the pathogen and distinguishable from “self” (Janeway, 1989). In RNA viral infections, PAMPS are features that are not usually present in cellular RNA, such as double-stranded RNA (dsRNA) or the presence of 5’triphosphate (5’ppp) and 5’diposphate (5’pp) groups (Killip et al., 2015). Pattern recognition receptors (PRRs) present in the host recognize these PAMPs and bind to them within infected cells. There are different classes of PRR that are involved in the activation of IFN pathways; the Toll-like receptor (TLR) family, RIG (retinoic acid inducible gene-) I-like helicase (RLH) receptors, nucleotide oligomerization domain-like receptors (NLRs) and cytosolic DNA sensors. The role of a certain PRR depends on the cell type and the nature of the viral stimuli; TLR and RLRs mainly respond to RNA viruses, whereas DNA sensors defend against DNA viruses. Upon recognition of PAMPs on the viral molecule, PRRs present at the cell surface or intracellularly in endosomes, signal to the host the presence of an infection. Intracellular signaling cascades are then activated which ultimately result in the expression of antiviral genes that orchestrate the early host innate response to infection (Mogensen, 2009).

## Toll/Interleukin-1 (IL-1) Receptor-Mediated Interferon Production

TLRs are the largest and most widely studied class of PRRs. They are glycoproteins that possess an extracellular domain containing leucine-rich repeats (LRRs) (which recognize a variety of ligands and bind to them), a transmembrane helix and a cytoplasmic signaling Toll/interleukin-1 (IL-1) receptor homology (TIR) domain (O’Neill and Bowie, 2007). TLRs are localized at the cellular or endosomal membranes such as the endoplasmic reticulum, lysosome or endosome where they recognize PAMPs via their LRR domain and transduce signals to the intracellular environment through the TIR domain. Antigen presenting cells such as macrophages and dendritic cells are arguably the most valuable cells that express TLRs; nevertheless, TLRs have been identified in most cell types (Iwasaki and Medzhitov, 2004). There are 10 different types of TLR identified in humans (TLR1-TLR10) which can be divided into subgroups dependent on the PAMPs that they recognize from different pathogens such viruses, bacteria, protozoa and fungi (Akira et al., 2006). TLRs signal via the recruitment of specific adaptor molecules such as MyD88 and TRIF (Kawasaki and Kawai, 2014) which lead to the activation of the transcription factors NF-κB, IRF3 and IRF7, ultimately leading to IFN and cytokine production. Three signaling pathways have been identified as essential in facilitating TLR-induced responses; those of mitogen-activated protein kinases (MAPK), NF-κB and IFN regulatory factors (IRFs). Despite NFκB and MAPK playing vital roles in the inflammatory response, IRFs are considered the most essential components required for IFN production. TLR1, TLR2, TLR4, TLR5 TLR6 and TLR10 located on the cell surface, recognize lipids and proteins and signal via the MyD88 pathway. Whereas TLR3, TLR7, TLR8, and TLR9 are intracellular, located at the endosome where many viruses un-coat their genomes and enter the cytoplasm. These intracellular TLRs recognize the nucleic acids; dsRNA, single-stranded RNA (ssRNA) and DNA, respectively and signal via the TRIF pathway (Majer et al., 2017). Upon engagement with ligands MyD88 activates MAPK and NF-κB which translocates to the nucleus, resulting in the synthesis of inflammatory cytokines such as TNFα. Alternatively, the TRIF pathway signals via the Toll-IL-1R domain-containing adaptor, inducing a signal cascade in which IRF3 or IRF7 translocates to the nucleus to induce the synthesis of type I IFNs; IFNα and IFNβ (Bagchi et al., 2007). TRIF is also able to activate through NF-κB leading to the production of inflammatory cytokines. TLR4 is unique in its ability to activate both the MyD88 and TRIF pathways (Hoebe et al., 2003).

## Cytosolic PRR Signaling

Although TLRs play a very significant role in sensing viral RNA at the cell membrane and in endosomes, it is apparent that additional sensing mechanisms must also take place inside of the cell, within the cytosol to aid in the contribution to host anti-viral defences.

### Retinoic Acid-Inducible Gene-I Like Helicase Receptor-Mediated Interferon Production

RLH receptors are critical components of the anti-viral defence pathway. They are present in almost all cell types and consist of three RNA helicases; retinoic acid-inducible gene (RIG-I), laboratory of genetics and physiology-2 (LGP-2) and melanoma differentiation associated gene (MDA5) (Thompson et al., 2011). RLHs recognize intracellular RNA that is introduced to the cell cytosol in a viral infection or is produced during viral replication. RIG-I senses 5’triphosphorylated uncapped ssRNA or short dsRNA and MDA5 recognizes long dsRNA. Upon recognition of this cytosolic viral RNA both RIG-I and MDA5 bind an adaptor protein called MAVS, which then initiates a signal transduction cascade. This in turn, leads to the activation of transcription factors such as NF-κB and IRFs causing IFN and inflammatory cytokine production. LGP2 has not been found to signal when interacting with viral RNA but seems to negatively regulate the other two RLHs in an unknown manner (Pippig et al., 2009). RIG-I and MDA5 consist of tandem N-terminal caspase activation and recruitment domains (CARD) in addition to a DExD/H box RNA helicase domain which has ATPase activity and a C-terminal repressor domain (CRD) (Thompson et al., 2011). Whereas LGP2 only consists of the RNA helicase domain as it lacks the two N-terminal CARD domains. RIG-I and MDA5 seem to recognize different classes of RNA viruses. As mentioned above, RIG-I is able to recognize viral RNA by interacting with 5’ triphosphate “blunt ends” of RNA, whereas MDA5 PAMPs are unclear but apparently discriminate between self and non self RNA based on their sequence length (Thompson et al., 2011).

### DNA Sensor-Mediated Interferon Production

TLR9 is primarily expressed in dendritic cells and B cells and is known to stimulate type I IFN production in response to foreign non-methylated CpG DNA in endosomes, via interacting with MyD88 to activate MAPK and NF-κB (Barber, 2011). There are many proposed cytosolic DNA sensors in addition to TLR9, including DAI/ZBP1, IFI16 and RNA Pol III. However, the two majorly characterized receptors consist of AIM2, a member of the PYHIN family, and cGAS (Xia et al., 2016). Upon stimulation in the cytosol by viral dsDNA, these sensors use the adaptor protein called stimulator of IFN genes (STING) to activate TBK1 and IRF3 and trigger type I IFN production. The signaling pathway linking DNA sensors to downstream effectors and TBK1 currently remains poorly characterized (Thompson et al., 2011). Other important pathways that are also activated by this method of intracellular DNA recognition include the inflammasome pathway, autophagy and cell death (Paludan and Bowie, 2013).

## The Interferon Response

Once activated, IFNs are secreted by infected cells where they enter the extracellular space and work to induce an antiviral state in the infected or neighboring cells in an autocrine or paracrine manner, respectively. In addition, they can also moderate innate immune responses to permit the action of antigen presentation and NK cells, but without the detrimental overactivation of pro-inflammatory pathways. Furthermore, IFNs can activate the adaptive immune system to induce specific T and B cell responses to the invading pathogen (Ivashkiv and Donlin, 2014). There are three types of IFN that are categorized based on which receptor they interact with; type I IFN interacts with receptors located in fibroblast cells, type II IFN receptors are located on endothelial cells and type III IFN receptors are largely found in immune cells. IFN receptors are comprised of heterodimers of two proteins with transmembrane domains, from which they recruit specific protein kinases that activate upon extracellular IFN binding to their cognate receptors (Sen, 2001). Activation involves dimerization of the IFN receptors which then activate downstream signaling pathways. Both type I and type III IFNs activate the JAK-STAT pathway. Type I and III IFNs both phosphorylate STAT1 and STAT2 and recruit IRF9 to form the ISGF3 complex which translocates to the nucleus and binds to the IFN-stimulated response element (ISRE) sequence. This induces the transcription of over 1,000 genes that act as antiviral defences, known as ISGs. Type II IFNs act differently to induce ISG transcription via the formation of a phosphorylated STAT1 homodimer, known as the gamma activation factor (GAF) complex, which translocates to the nucleus and binds the IFGAS sequence. The ISGs produced by all three IFN types act as antiviral effectors to protect host cells against pathogenesis via various methods, including; inhibition of translation, inhibition of viral entry, sequestration of viral mRNA translation, and the inhibition of viral transcription.

## Bats as Viral Reservoirs

Bats are grouped in the order Chiroptera and are one of the most abundant and geographically widespread vertebrates on Earth. The order Chiroptera can be subdivided into Yinpterochiroptera (consisting of megabats and some microbat species) and Yangochiroptera (composed of the remaining microbat families). Within these suborders high amounts of diversity are observed between bat size, ecological niches, diets, and morphology (Lei and Dong, 2016). There are over 1300 species of bats, representing more than 20% of all mammals on earth, deeming them the second most diverse mammalian group, after rodents (Wang and Anderson, 2019; Devaux et al., 2018). Bats are an important reservoir of zoonotic viruses and have been shown to harbor many viruses that are highly pathogenic to humans such as; rabies virus, Hendra virus and Nipah virus. They also harbor coronaviruses that are believed to have caused disease in humans after spillover events into intermediate hosts, including severe acute respiratory syndrome (SARS), Middle East respiratory syndrome (MERS), and the recently emerged 2019 novel coronavirus (CoVID-19) (Devaux et al., 2018). Similar genomes of these medically important viruses have been detected in bats where occasionally, only viral antibodies are identified in various bat species. As expected in reservoir hosts, despite harboring many viral species, bats rarely exhibit signs of disease. However, there are exceptions to this generalization as some viruses are known or suspected to kill bats including most, if not all, lyssavirus species, Tacaribe arenavirus, and Lloviu cuevavirus (Negredo et al., 2011; Cogswell-Hawkinson et al., 2012). Furthermore, the virus known as Zwiesel bat banyangvirus has been recently discovered to kill bats from northern Germany (Kohl et al., 2020).

Bats are able to transmit viruses to humans in “spillover events” either directly or via an intermediate host. Significantly, the recent emergence of COVID-19, appears likely to have originated in bats and has entered human populations via an unknown intermediate host, likely traded in the Wuhan market at the epicentre of the outbreak. COVID-19 is the third identified highly pathogenic coronavirus to enter human populations (Zhou et al., 2020). Sequence comparison and evolutionary analysis of the SARS-CoV-2 genomes obtained from COVID-19 patients have revealed a high sequence similarity (~96%) with β-coronavirus of bats origin (Bat_CoV_RaTG13) (Zhou et al., 2020). This suggests that the bat and human COVID-19 share the same viral ancestor, although bats were not traded at the seafood marked in Wuhan (Wu et al., 2020). It is thought that certain stressors on bats such as disease or habitat loss for example, cause disruption of the viral-immune co-existence they possess. Upsetting this equilibrium therefore permits the multiplication of the virus, increasing its virulence and allowing transmission into other hosts (Rocha et al., 2020).

Bats possess unique characteristics that are distinguishable from other mammals which could possibly underlie their ability to harbor many viruses without showing clinical symptoms. However, it should also be understood that most other mammals are also able to harbor many viruses without exhibiting symptoms and it is the bats apparent unique immune characteristics that are important here in allowing them to harbor viruses. Bats are the only mammal capable of powered flight, allowing certain species to travel over large geographical distances during seasonal migrations and in pursuit of food where they may mix with other bat populations and hence contribute to the spread of viruses (Holland, 2007). Bats also have extremely long lifespans, Microchiroptera (microbats) for example, have life spans of around 25–35 years which is a longevity rarely seen in other mammals with similar body mass to metabolic rate ratios (Calisher et al., 2006) These features may potentially allow bats to host and spread viruses for longer durations and hence warrant future investigations. Moreover, the large population densities of bats and their mating behaviors, are extremely likely to increase the transmission of viral infections between individual bats and can henceforth also increase their transmission rate to other potential hosts.

These observations and studies highlight the need to explore diversity in the innate antiviral immune responses within this fascinating order of mammals and the seemingly unusual abilities of bats to control virus induced pathologies. In this section, bat IFN systems are discussed with the primary focus being the black flying fox (*Pteropus alecto*) and what we can learn from this bat as a model species.

## The Bat Interferon System

### Background

The black flying fox is most commonly used as the model species when studying bats and has therefore been used in most of the previous immune studies conducted on bats. Studies in the black flying fox and a few other bat species have identified many factors of antiviral immunity known in humans to be conserved in bats, including PRRs, IFNs, IFN receptors, and the ISGs they induce (De La Cruz-Rivera et al., 2018). Mutations in viral RNA species often result in the virus becoming biochemically “optimized” to exist in a particular host. It is therefore conceivable that RNA viruses have evolved alongside their bat hosts, allowing both to co-exist with each other. This could suggest why bats are able to harbor many different RNA viruses without exhibiting pathology. It is also suggested that bats may have adapted certain immune mechanisms that also aid in the establishment of a unique host-virus relationship (O’Shea et al., 2014), although the mechanisms underlying disease tolerance in bats remains largely unknown. One hypothesis proposed by (Baker and Zhou, 2015) suggests that bats are able to control viral replication early on in the immune response, via antiviral mechanisms and the stimulation of ISGs. There are two types of immunity shown in bats; innate and adaptive, innate immunity is the first line of defence against viruses and primes the adaptive response against the virus. This review is focusing largely on the IFN arm of the innate immune response, which are the first cytokines to respond to viral infection in bats.

### Bat Pattern Recognition Receptors

As previously discussed, different PRRs have been identified in humans that appear to show certain homology to those found in bats. TLRs have been characterized from the black flying fox and the fruit bat Leschenault’s rousette (*Rousettus leschenaultia*). Studies by Cowled et al. (2011a) have identified TLRs 1–10 in the black flying fox in addition to the nearly intact pseudogene TLR13 which is lacking in humans and most other mammals, but has only been previously identified in rodents (Zhang et al., 2013b). The TLR13 described in the black flying fox lacks a suitable start codon and contains 3 in-frame stop codons, suggesting that the pseudogene has recently been inactivated. Notably, the TLRs that are associated with nucleic acid sensing (TLRs 3, 6, 8, and 9), appear conserved between humans and bats, indicating the homology of bats viral recognition mechanisms with other mammals. Genomic analysis of TLR7 has indicated that it had evolved quicker in bats than other mammals; however, its function in bats still remains largely unknown. Baker and Zhou (2015) have suggested that the coevolution of viruses and bats may have caused changes in TLR7 that affect ssRNA recognition in bats.

Three types of RLH recognize viral RNA and DNA in the cell cytosol of most eukaryotic cells. Homologous to human RLHs; RIG-I, MDA5, and LGP2 have been identified in the black flying fox via transcriptome analysis (Cowled et al., 2011b). Bat RLHs show similarity to humans in their structure and expression (Baker and Zhou, 2015). Studies conducted by Cowled et al. (2011b) have proved that upon stimulation with synthetic dsRNA, all three helicases were upregulated in bat kidney cells, suggesting a functional homology in viral recognition between bats and other mammalian species.

DNA sensors identified in humans include AIM2 and IFIT16 which are associated with inflammasome assembly in addition to TLR9 and cGAS that are involved in IFN expression. There is currently little known about bat DNA sensors; however, findings of a recent study showed that the most positively selected genes in bats are involved in innate immunity and the DNA damage pathway (Zhang et al., 2013a; Hawkins et al., 2019). NLRP3 is one of these genes, as identified in the black flying fox, to act as an inflammasome sensor via the activation of caspase-1, cleaving IL-1β, and IL-18. In humans, the inflammasome can also be activated by non-NLR proteins called AIM2 and IFIT16, belonging to the PYHIN family. However, these appear to be absent in all bat genomes sequenced to date, suggesting that bats have a lowered DNA-triggered inflammasome response to viruses (Ahn et al., 2016). In addition, TLR9 appears to be more highly expressed in bats than its other mammal counterparts. These discoveries could suggest that bats have evolved a unique IFN response in adaption to flight, which other mammals do not possess (Xie et al., 2018).

### Bat Interferons: Production and Receptor Interactions

The IFN response is a key part of the innate immune system, acting as the first line of defence against viral infection. Type I and type III IFNs are induced in vertebrates in response to viral infection and are essential in establishing an antiviral state of host cells by the transcriptional activation of ISGs. Type I IFNs have been identified in five different bat species (Baker and Zhou, 2015) and are secreted from cells in response to viral infection, binding to IFN receptors to activate ISGs. The first transcriptome analysis of IFNs in any bat species was conducted by Zhou et al. (2016) in the black flying fox. They identified that the black flying fox consisted of only 10 type I IFNs, including three IFNα genes and thereby deduced that the black flying fox possesses a restricted type I IFN locus containing fewer IFN genes than other mammals. Interestingly, in contrast, IFNδ and IFNω genes appear expanded in the black flying fox, when compared to other mammals (Baker and Zhou, 2015). The contraction of the black flying fox IFNα locus together with its expanded IFNδ and IFNω genes, has not been observed in other species and could offer evidence for the unique host-virus symbiosis found in bats. It is feasible that the overexpression of IFNδ and IFNω compensates for the lack of IFNα response. In addition to the black flying fox, studies on other bat species have identified the IFNα locus to contrastingly appear expanded in the Egyptian fruit bat (*Rousettus aegyptiacus*), the greater flying fox (*Pteropus vampyrus*), and the little brown bat (*Myotis lucifugus*) collectively (**Table 1**) (Kepler et al., 2010; Pavlovich et al., 2018). Type III IFNs have recently been identified in bats and found to signal through the same pathway as type I IFNs but via a different receptor complex. Type III IFNs that have been identified in the black flying fox by Zhou et al. (2011a) include; IFNλ1 (IL-29) and IFNλ2 (IL28B), which appear to show homology to other mammals with similar loci and sequence length, indicating a functional conservation (Virtue et al., 2011).

**Table 1:** Nature of IFNs expression in currently studied bats species.

Bat Species	Type I IFN	Type III IFN	References
Australian Black Flying Fox *(Pteropus alecto)*	• Constitutively expressed IFNalpha • Contracted locus • No upregulation with Tioman virus • Antagonized by henipavirus	• Induced after viral infection with Tioman virus • Antagonized by henipavirus	Zhou et al., 2016 Zhou et al., 2011a
*Egyptian Fruit Bat (Rousettus aegptiacus)*	• No constitutive expression observed • Induced after viral infection • Extensive expansion of IFNw genes	Not determined	Pavlovich et al., 2018 Omatsu et al., 2008
*Common vampire bat (Desmodus rotundus)*	• Induction after polyI:C stimulation • Induction of selective IFN-stimulated genes	Not determined	Sarkis et al., 2018
Daubenton’s bat *(Myotis daubentoniid)*	• Induction of IFN-stimulated genes • Induction of IFNs by virus and polyI:C	Not determined	Holzer et al., 2019

Little is known about IFN production and the signaling pathways involved in bats as few studies have been conducted. Early studies by Stewart W.E II (1969) were the first to prove that IFN signaling pathways exist and are functional via the stimulation of bat cells with synthetic dsRNA (poly I: C) and lipopolysaccharide (LPS). Other studies that were conducted on different types of bat cells, appear to contradict each other. Splenocytes, which are immune cells, were taken from the black flying fox and experimentally infected with the bat paramyxovirus Tioman virus and resulted in the downregulation of type I IFNs, but also the upregulation of type III IFNs (Zhou et al., 2011b). These results could imply that this upregulation of type III IFNs could play a role in bats inimitable abilities to coexist with viruses. However, the roles of IFN-inhibitory proteins of these viruses were not investigated. In another experiment, infection of fibroblast cells from the black flying fox with henipavirus antagonized both type I and type III IFN production. The different results observed between the infected bat cells may be due to the immune specialty of splenocytes, giving them alternative IFN production mechanisms to fibroblast cells (Baker and Zhou, 2015). IFN production in bats generally appears similar to that of humans and other mammals. (Zhou et al., 2014) characterized all IRF family members from the black flying fox genome and found the IFNβ promotor region contains conventional IRF3 and IRF7 binding sites. IRF and NF-κB binding sites have also been identified in the promotor regions of IFNκ and IFNω in the serotine bat (*Eptesicus serotinus*) (He et al., 2014). IRF7 in humans is restricted to certain tissues, however an interesting finding by (Zhou et al., 2014) found that IRF7 mRNA in cells of the black flying fox is more broadly distributed across tissues compared to mice and humans and is also constitutively expressed. This broad IRF7 expression may contribute to the ability to activate IFN responses in multiple tissues and cells and thereby respond more rapidly to infection. However, this has only been identified in a single bat species and hence requires further research to explore the IRF7 expression in other bats to determine whether it is a feature identifiable in all bat species.

The type III IFN receptor (IFNλR) has been well characterized in the black flying fox (Zhou et al., 2011b). It was revealed that IFNλR is transcribed in virus-infected bats, regardless of the suppression of type I IFNs. The IFNλR in the black flying fox is comprised of two genes; IFNλR1 and IL10R2 and appears homologous to the type III receptor found in humans and other mammals. IFNλR is widely distributed at the tissue and cellular level, present in both immune and epithelial cells, where it is receptive to IFNλ treatment and therefore presents as a functional receptor (Zhou et al., 2011b). This distribution is consistent with previous findings that suggest type III IFNs play a more vital role in bat antiviral immunity, as type III IFNs are upregulated, while type I IFNs are simultaneously downregulated upon viral invasion. Little is known about the type I IFN receptor in bats as no experimental studies have been carried out. However, genomic analysis has been conducted on certain bat species and found that the IFNAR1 gene has undergone positive selection in the vesper bat species *Myotis davidii*, but interestingly not in the black flying fox (Zhou et al., 2011b) and the consequences of this on their immune response is currently unknown.

**Figure 1:**
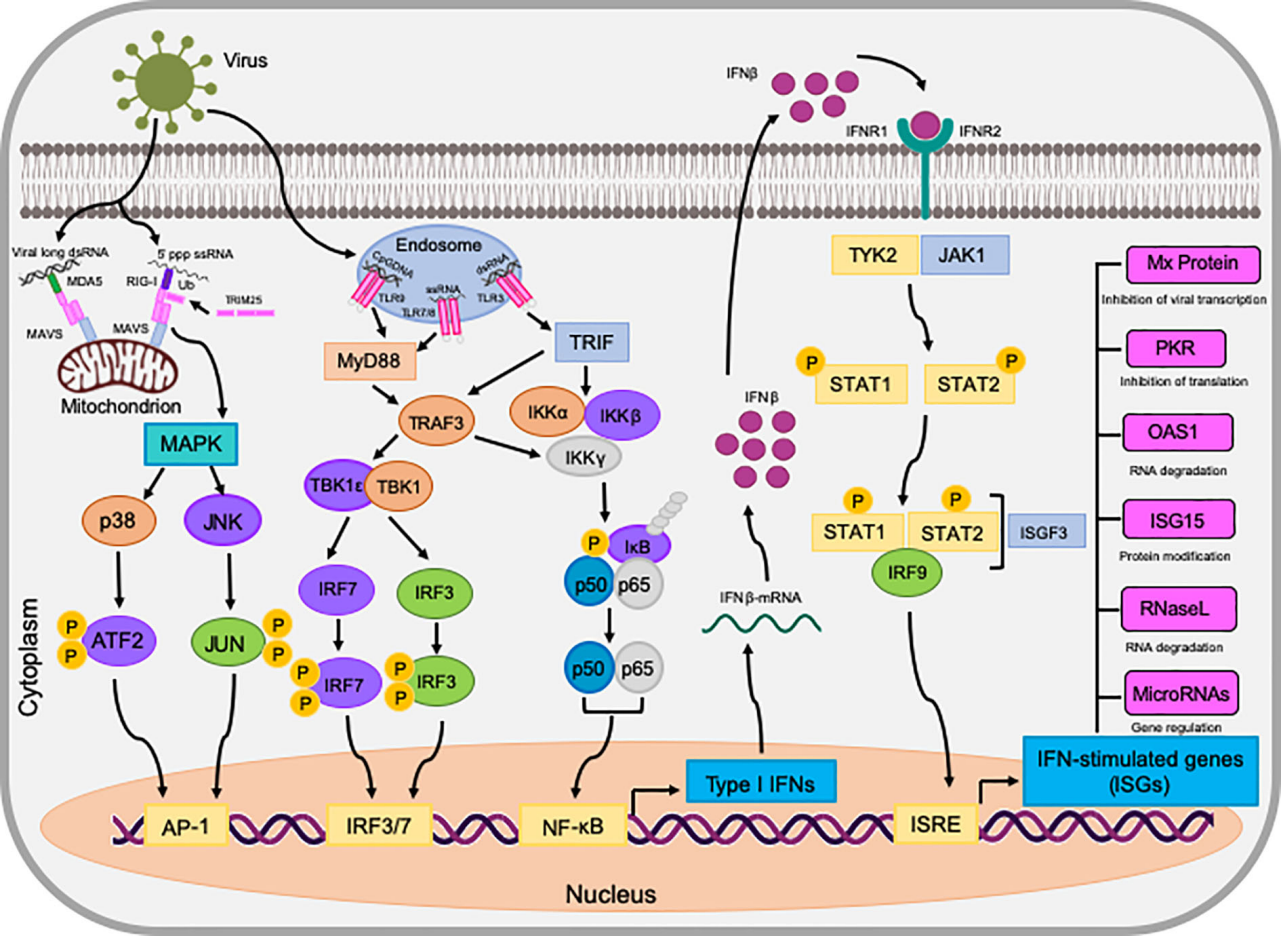
The induction of IFNs and the antiviral state they exhibit in bat (*P. alecto*) cells. dsRNA is detected by either RIG-I/MDA5 or TLRs on endosomes, initiating downstream signaling via MAVS, TRIF, and TRAF3. These adaptors activate the transcription factors IRF3, IRF7, NF-κB, and AP-1 via the assembly of multi-protein complexes. Upon activation, these transcription factors translocate to the nucleus and stimulate the transcription of interferons, such as IFNβ. IFNβ then binds to its cognate receptor complex IFNAR via autocrine and paracrine manners to activate the JAK-STAT pathway. This terminates at the activated ISGF3 transcription factor which in turn, translocates to the nucleus and initiates the transcription of genes in ISRE promoters known as ISGs. ISGs then act in a multitude of ways to establish an antiviral state in the cell against invading pathogens.

Once type I and type III IFNs have bound to their cognate receptors, they activate the same signaling pathway, called the JAK-STAT pathway, which ultimately leads to the activation of antiviral effectors and ISGs, as detailed in **Figure 1**. Little work has been directed toward identifying IFN signaling in bats, however experiments by (Brzozka et al., 2006) found that stimulation of bat cells with human IFNα, resulted in the translocation of STAT1 into the nucleus, similar to the activation found in other mammal species. Therefore, it can be deduced that bat IFN signaling downstream of receptors appears to be comparable to other mammals.

## Immune Features in Bats

The black flying fox can harbor certain viruses without showing signs of disease but can still transmit the virus to other mammals and humans in spillover events, in which the virus can cause pathology in the infected host. It is therefore imperative that this ability as to how these bats remain unharmed while harboring these viruses is unearthed. It is suggested that the evolution of many characteristics of bats such as being the only flying mammal, having long life spans, their nocturnal abilities and reproductive mechanisms, may all contribute to the hypothesized uniqueness of their immune response that has not been observed in any other species. This section will discuss comprehensively the different plausible traits that bats possess, focussing largely on the black flying fox, that may prove advantageous in coexisting with viruses.

### Unusual IFNα Expression in Bats

Research by (Zhou et al., 2016) was the first to characterize the type I IFN locus in the black flying fox and compare it to other species. They found that bats contain fewer IFN genes than any other known mammal and found that the black flying fox only possesses three IFNα genes. In addition, they revealed that IFNα genes are constitutively expressed in unstimulated bat cells and tissues where their level remains unaffected by viral infection. Infection of *P. alecto* kidney PaKiT03 cells with two bat-borne viruses (Hendra virus and Pulau virus) caused no change in the constitutive IFNα expression pattern. This expression has not been observed in any other species which suggests its significance in the bats ability to coexist with viruses. To ensure that the constitutive IFNα expression was unspecific to the black flying fox, they conducted the same studies using the lesser short nosed fruit bat (*Cynopterus brachyotis*) and found similar results of continually high IFNα expression across all tissues, regardless of viral stimulation (Zhou et al., 2016). Functionality of the black flying fox IFNα proteins were assessed using transfection experiments in human HEK293T cells, which displayed successful induction of ISGs in all three black flying fox IFNα proteins (Zhou et al., 2016). Findings suggest that IFNα is not upregulated in response to viral dsRNA sensing in these bats, but instead the high baseline levels of IFNα means that it can still be detected in the absence of immune stimulation. Contraction of type I IFNs in the black flying fox and the differences in their expression patterns, is consistent with the “less is more” theory that natural selection can produce mutations that favor fewer functional genes, but with advantageous consequences to the host (Olson, 1999). Additionally, Zhou et al. (2016) have determined that bats use fewer IFNα genes to perform functions, in comparison to IFNαs identified in other species, by using a system that is constitutively primed to respond to viral infection. Research by Shaw et al. (2017) provides further supporting evidence to the constitutive expression of IFNα as they identified the basal transcription level of type I interferome in both megachiropteran and microchiropteran cells to be significantly higher than the other species studied (**Table 1**). Despite possessing fewer IFNα genes, the ubiquitous and constant expression of IFNα in some bats may provide them with an effective system for controlling viral replication, allowing them to exist in a constantly active antiviral state. It can be concluded that the observable antiviral mechanisms in bats that differ between species can is likely to have arisen via convergent evolution and tolerance mechanisms identified in the black flying fox should not be generalized across all bat species. This highlights the need for further exploration of IFNα expression in further bat species. Furthermore. the contrasting expression patterns of IFNs already identified in some bat species highlights the inter-species diversity in bats when mounting immune responses against pathogens and eludes to the existence of additional features between different bat species.

### IRF7 Distribution

Studies by (Zhou et al., 2014) have used the black flying fox as a model species to explore the role of the IFN system in the regulation of viral replication in bats. Bats appear to show a higher expression and wider distribution of type III IFN receptors than type I, suggesting that type III IFNs have a key role in the black flying fox’s antiviral immunity (Zhou et al., 2011b). Significant evidence was provided by (Zhang et al., 2013b) that backed the positive selection of genes in the IFN pathway, including TLR7, TBK-1, IFN-ϒ, ISG15, and RIG-1 which may be due to the co-evolution of bats and viruses, and may assist in the ability of these bats to asymptomatically coexist with viruses. As IRF7 is a master regulator, central to the IFN-dependent immune response, Zhou et al. (2014) performed sequence and functional analysis of the black flying fox IRF7 to provide evidence for the conserved IRF7 functionality observed in bats, despite its sequence variation. IRF7 is expressed in low levels in most cell types in other animals but is highly expressed in immune cells such as dendritic cells and is induced in type I IFN mediated signaling in these cells via the activation of TLR7/9 and the MyD88 dependent signaling pathway (Ning et al., 2011). Results from Zhou et al. (2014) have confirmed that the activity of bat IRF7 is conserved but found it to have a wider tissue distribution and unique expression pattern in both immune and non-immune cells. It is hypothesized that the broad distribution of IRF7 may increase the ability to activate the IFN response in a wider range of tissues than found in other mammals, enhancing bats antiviral immunity. There is a lack of data on the tissue distribution of IRF7 in other bats and also in other mammals except from human, mice and horses; however, the broad distribution of IRF7 in bats has also been observed in at least five species of fish and was hence hypothesized to play a key role in fish antiviral immunity (Zhang et al., 2003). Zhou et al. (2014) suggest that further analysis of cell types responsible for the IRF7 expression in bats is required but suggested that the constitutively expressed IRF7 in a broad range of tissues may result in a faster and stronger IFN production upon viral infection (Ning et al., 2011). Upon analysis of the IRF7 protein sequence in the black flying fox, there is an apparent deletion at around 260 amino acids, when compared to its human counterpart. The deleted section lies between two domains; the constitutive activation domain (CAD) and the virus-activated domain (VAD). Evidence suggests that this could be an evolutionary deletion attributed to the ability of bats to co-exist with viruses, allowing the IRF7 to remain active but functionally different to its human counterpart. However, similar to IFN expression diversity in the black flying fox and the Egyptian fruit bat, there may be species level disparity, and this thereby warrants future investigations.

### Positive Selection of Bat IRF7 and Antiviral Responses

IRF3 and IRF7 are critical transcription factors in driving the expression of IFNs (Randall and Goodbourn, 2008). Sequence analysis of putative IRF3 sequences reveals evolutionary differences among bats when compared to other mammals. A recent functional study in the big brown bat (*Eptesicus fuscus*) indicated a mammalian-like MERS or dsRNA-induced stimulation of IFN production (Banerjee et al., 2019). In contrast, silencing of IRF3 in the big brown bat resulted in suppressed IFN activation with similar stimuli. However, the molecular mechanism of this induction remains elusive. Recently, computational analyses of bat IRF3 revealed a highly conserved serine residue at position 185 (S185) in 7 of the 11 examined bat species. Replacement of S185 with D185 in bat IRF3 conferred an enhanced protection against model vesicular stomatitis virus. Interestingly, substituting the leucine residue of human IRF3 with corresponding serine residue from bat IRF3 significantly enhanced antiviral protection in human cells (Banerjee et al., 2019; Banerjee et al., 2020). These insights support the notion that bats have acquired multiple adaptations in their antiviral immune responses to co-exist with pathogens. While these initial studies are projecting an interesting side to the bat’s antiviral responses, it remains to be explored if this positive selection is species-specific and its biological relevance against emerging and bat-borne viruses.

### Dampened Nucleotide Oligomerization Domain-Like Receptor Family Pyrin Domain Containing 3 Inflammasome Response

**Figure 2:**
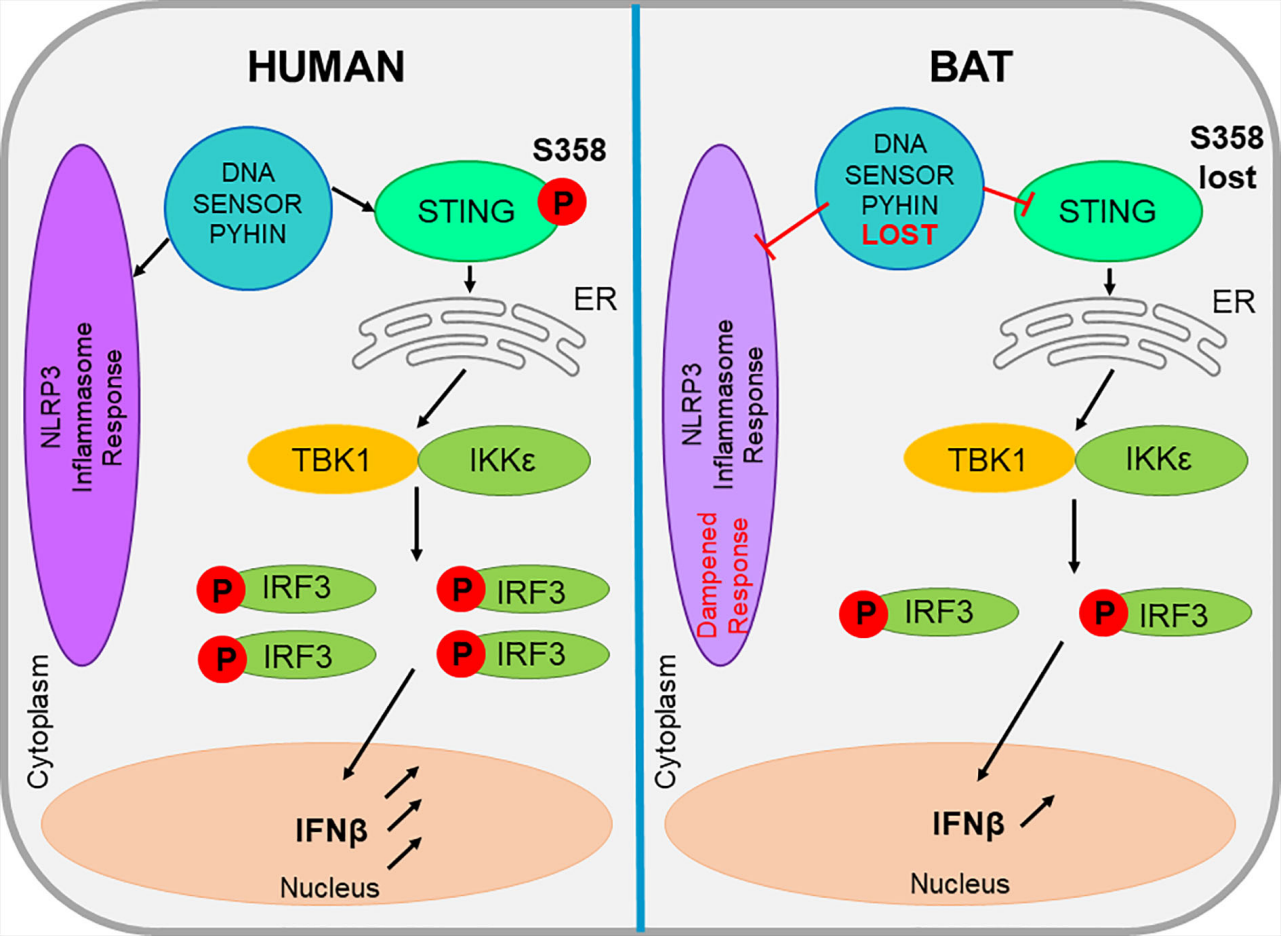
The comparison of bat and human STING and inflammasome activities within a cell. The evolutionary loss a DNA sensor belonging to the PYHIN gene family leads to a dampened NLRP3-mediated inflammasome response. This loss also impacts the action of STING, which in bats lacks a serine residue, reducing its functionality. These factors all appear unique to bats and all contribute to the reduced level of IFN produced in bat cells.

The NLR family pyrin domain containing 3 (NLRP3) inflammasome sensor has been proven central to age related and viral-induced inflammation in humans and other mammals.NLRP3 is vital to the inflammasome of cells and its role is to recognize cellular stresses such as mitochondrial damage or oxidative stress, in addition to bacterial or viral infections. NLRP3 is known to respond to a myriad of viruses, including bat-borne viruses such as rabies and influenza (Ahn et al., 2019). Until recently, nothing was known about NLRP3-mediated inflammation in bats, but it was hypothesized that despite the NLRP3 inflammasome existing as a central player in viral infection in bats, it differs between bats and other mammals. Leading research by Ahn et al. (2019) demonstrated an overall dampened activation of NLRP3 in bat primary immune cells, when compared to their human or murine counterparts (**Figure 2**). Bats were shown to display a dampened host inflammasome response to both viral and bacterial infections and cellular danger signals. (Ahn et al., 2019) tested bat cells by infecting them with three different zoonotic RNA viruses and found that viral loads remained unaffected in the bat primary immune cells. This was due to the dampened transcriptional priming and decreased functional capacity of bat NLRP3. Upon testing wild and experimentally infected bats, it was evident that they were able to tolerate viral diseases, even with a high viral load present in the host. Evidence shows that bats have naturally dampened stress-related and pathogenic sensor induced responses which coincides with their ability to exist as asymptomatic viral reservoirs (Ahn et al., 2019). The dampened NLRP3 response that has been identified in bats, supports the theory that they possess an enhanced immune tolerance, as appose to an enhanced antiviral defence (Ahn et al., 2019). Therefore, this aids in the explanation of inflammasome significance in disease tolerance in bats, in contrast to the pathogenesis observed in spillover hosts.

### Absence of Pyrin and HIN Domain Gene Family

In addition to the positively selected NLRP3 inflammasome sensor, the entire pyrin and HIN domain (PYHIN) containing gene family appears evolutionarily lost in bats, suggesting a dampened DNA-triggered inflammasome response as shown in **Figure 2**. The PYHIN gene family are important immune sensors of intracellular and foreign DNA and activate the immune response. They are the only DNA sensors capable of activating the inflammasome (Hartlova et al., 2015). Previous genome analysis of two bat species (*P. alecto* and *M. davidii*) revealed the absence of the PYHIN gene family in both species. Studies by Ahn et al. (2016) further these findings and analyze 10 different bat species, which covered four of the five major bat lineages and confirmed the complete loss of this gene in all species, despite the presence of the PYHIN locus. The only minor discrepancy in this study was the identification of a truncated AIM2 gene in the Parnell’s moustached bat (*Pteronotus parnellii*,) which is thought to be where the bat PYRIN sequence clustered with AIM2, indicating the presence of a functional AIM2 gene in the bat common ancestor that was lost during evolution. All other major groups of placental mammals possess at least one gene member, whereas most bats appear to have lost the entire PYHIN gene family as a loss of function evolutionary event (Ahn et al., 2016). The unique absence of PYHIN genes in bats suggests it may be an important adaptation that is possibly induced by flight and affects DNA sensing and inflammasome activation (Ahn et al., 2016). Bats are the only mammals capable of flight, which is considered metabolically costly; however, bats have the ability to increase their metabolic rate up to 34 times their resting rate to compensate for this (Thomas and Suthers, 1972). The exclusive loss of PYHIN in bats suggests an important adaptation for flight. Despite still possessing other cytosolic DNA sensors, such as cGAS, STING, and ZBP1, PYHIN is the sole activator of the inflammasome (Dempsey and Bowie, 2015). Therefore, its deletion may enable some bats to limit excessive inflammation activation and in turn, regulate the type I IFN response which is normally triggered by PYHIN proteins recognizing the DNA damage and release of self-DNA from the metabolic activities of flight. There is a likelihood that the increased exposure of bats to many zoonotic RNA and DNA viruses when compared to other mammals that do not cover large distances, may be the evolutionary driver of PYHIN loss, or contrastingly, the loss of PYHIN may allow for this bat-viral co-evolution (Ahn et al., 2016). The loss of PYHIN in bats is also hypothesized as a basis for the long lifespans that bats exhibit.

### Dampened Stimulator of Interferon Genes -Dependent Interferon Activation

Cytosolic DNA either produced from flight or viral infections imposes selective pressures on bat DNA sensors such as the PYHIN gene family mentioned previously. This results in a dampened sensing mechanism and downstream IFN production to avoid overreaction on a regular basis during flight or due to viral co-existence (Xie et al., 2018). While bats detect and respond to RNA viruses, studies have shown that the response of bats to DNA infections is dampened. Along with the absence of the PYHIN gene family, the ability of STING, an essential adaptor protein involved in multiple DNA-sensing pathways to induce IFN expression, also appears dampened in bat cells. This can be attributed to the mutation of the serine residue at position 358 (S358) in STING. Xie et al. (2018) carried out sequence and functional analysis to identify the dampened, but not diminished, response of STING in bats (**Figure 2**) against herpes simplex virus (HSV) replication. By experimentally reversing the mutation, it was revealed that STING functionality was restored in the bat cells (Xie et al., 2018). The mutation of the serine residue at the phosphorylation site 358 in bats, resulted in the impaired ability to activate downstream IFNs (Liu et al., 2015) and was identified in every known STING protein of bat. This STING dampening explains the reduced ability of bat cells to detect self-DNA and exogenous DNA and is speculated to be a side effect to the evolution of powered flight in bats. During flight, the body temperature of bats can reach over 41°C. The elevated body temperature and high metabolic rates in bats can produce reactive oxygen species (ROS) which in turn cause DNA damage and the release of cellular DNA into the cytoplasm. Therefore, it is theorized to overcome this, bats have undergone a positive selection for various genes involved with DNA repair, which itself causes consequences for antiviral responses. It is unlikely however, that bats have lost all DNA-sensing machinery, as DNA viruses have been identified and isolated from many bat species (Banerjee et al., 2020). Future studies are required to understand the adaptations bat cells have evolved to sense DNA viruses while also limiting the detection of self-DNA. Theoretically, the evolution of flight in bats may have caused consequences for the immune response in minimizing the DNA damage associated with high metabolic rates. Also, exogeneous DNA sensing pathways may have been dampened to reduce self-DNA mediated immunopathology which in turn may have consequences for viral DNA detection in bats. Further research is also required to determine if bats have evolved novel mechanisms to sense and respond to exogenous and self-DNA (Ahn et al., 2016). STING remains conserved in every other mammal; therefore its dampened expression could possibly provide bats with an advantage that would likely be associated with flight and the ability to maintain an effective, but not over-reactive response to co-existing with some viruses (Xie et al., 2018).

### Immunogenetics and Viral Reservoir Potential of Bats

Recently, developments from the Bat1k project which started back in 2017 have now published six near-complete annotated bat genomes. Through screening, they investigated gene loss, gain and selection to identify novel genes that are likely associated with viral tolerance within bats. These results will hence prove extremely useful in expanding existing knowledge of bat immunology within this fertile field of research (Jebb et al., 2020). Upon performing unbiased genome-wide screens for gene changes within the six bat species, Jebb et al. (2020) identified nine genes that have undergone positive selection in the bat ancestor. Among these are the genes LRP2 and SERPINB6 which have known roles in hearing. The project deduced that LRP2 has an amino acid substitution that is only found in bat species that utilize echolocation, indicating its significance in hearing. Pteropodid bats are described as exhibiting a different amino acid within LRP2 and these bats do not have laryngeal echolocation. These results suggest that if these gene mutations are related to echolocation, then this would prove the origin of echolocation in bats with evidence for echolocation present in the bat ancestor that was subsequently lost in pteropodid bats (Jebb et al., 2020). More significantly to this review, the genome-wide screenings conducted by the Bat1K project also found that bat-specific selection had occurred on several genes that are related to immunity. These changes could potentially underlie the reasoning behind the unique tolerance of viruses identified in bats. Screening identified positive selection on genes such as INAVA, which has a role in enhancing NF-κB signaling in macrophages and the B-cell chemokine CXCL13. In addition to this analysis, this study also identified 10 additional genes that have undergone positive selection in the bat ancestral lineage, including some genes involved in immune regulation and NF-κB activation (IL17D and IL1B). Collectively, from the results of these studies, it was suggested that ancestral bats have evolved immunomodulatory mechanisms that permit a higher tolerance to pathogens in comparison to most mammals, supporting the overarching theory of this review that bats possess a unique immune response against viral infection. To further support findings, the Bat1k project also completed a second genome-wide screen, but this time aimed at identifying any inactivated genes or gene losses. 10 genes from the six genomes were inactivated, two of which normally have immune-stimulating functions, again related to NF-κB. LRRC70 normally enhances NF-κB activation and response to cytokines but is inactivated in these bat species (Wang et al., 2003). Moreover, the gene IL36G, encoding a pro-inflammatory interleukin that induces the NF-κB pathway (in addition to other inflammatory cytokines) was also inactivated in bats. Both screenings for positive gene selection and inactivation identified genes involved in NF-κB signaling, suggesting this apparent altered pathway may partake in the bats immune adaptations (Jebb et al., 2020).

The Bat1K project identified that within the bat lineage, the APOBEC gene family is expanded at its locus. This is significant as this gene encodes enzymes that edit DNA and RNA and are often induced by IFN signaling, hence deeming them useful in preventing viral infection. Thereby, expansion of this gene identified in these bat lineages is likely to contribute to the unique viral tolerance mechanisms that bats display (Jebb et al., 2020). Discovery of this evidence further supports the theory that bats can tolerate viral infection much more efficiently than other mammals due to their likely unique immune systems. Viral infections often leave traces within host genomes called endogenous viral elements (EVEs), so Jebb et al. (2020) deemed it important to also screen the six bat genomes to observe if the diversity of these elements differed within the bats compared to other mammals. The project took an approach to focus separately on non-retroviral EVEs and retroviral protein-coding EVEs respectively. Three non-retroviral EVE families were identified; *Parvoviridae*, *Adenoviridae*, and *Bornaviridae* which were found within the bats as well as other mammals, in addition to a partial filovirus EVE in Vespertilionidae. Retroviral protein-coding genes were identified to be highest from beta and gamma retroviruses as predicted by the project. It was also noted that in the genomes of some of the bat species, viral envelope-encoding DNA was identified that appeared more similar to alpharetroviruses, which had previously only been identified as endogenous avian viruses. Discovery of these alpharetrovirus elements within the bat genomes indicates that they must once have been infected by these viruses that appear originally avian in origin (Jebb et al., 2020).

## Interferon Stimulated Genes in Bats

Once the bat IFNs are triggered by a stimulus such as a virus, the transcription of hundreds of ISGs are stimulated. These ISGs are vital to the antiviral defence of bats, as they exert a multitude of antiviral mechanisms which collectively target almost any step in a virus life cycle (Schoggins and Rice, 2011). Despite the vast amount of information available about the IFN system of bats at the genomic level, there is still little known about the IFN-induced responses that are shaped by the transcription of ISGs. Overall, the bat interferome initially appears as standard in up-regulating core ISGs and possessing similar distributions of up or down regulated genes to other mammals along with having their own set of species-specific ISGs (Shaw et al., 2017).

### Conserved Interferon-Stimulated Genes

ISG expression is known to correlate with the establishment of an antiviral state of infected and neighboring cells. There are 50–1,000 ISGs identified in humans, based on cell type and duration of IFN treatment, yet it is currently unknown how many ISGs are induced in different bat cells and within different bat species (Banerjee et al., 2020). Recent research by De La Cruz-Rivera et al. (2018) studied ISG production in the black flying fox and identified that IFN signaling in this species consists of both unique and conserved ISG expression profiles. Numerous ISGs that are known to exist in humans and other mammals have been identified in the black flying fox upon stimulation with poly I:C, including protein kinase R (PKR), 2-5-oligoadenylaye synthetase 1 (OAS1) and orthomyxovirus-resistant gene 1 (Mx1 GTPase) (Zhou et al., 2013). These three ISGs are the most studied in bats and are representative of the major antiviral pathways that are induced in an antiviral response. The OAS1 gene promoter in black flying fox cells has two IFN-stimulates response elements (ISREs), in comparison to the one ISRE observed in the human OAS1 counterpart. This could possibly play an important antiviral role in RNA infections in the bat (Zhou et al., 2013). Studies that identified IFN-stimulated transcripts from the black flying fox found that over 100 genes are induced in response to IFNα, which have previously been identified as ISGs in other mammals, suggesting strong evolutionary conservation of ISGs in bats (De La Cruz-Rivera et al., 2018). The Papenfuss et al. (2012) have also identified further ISGs in the black flying fox via transcriptome analysis including; Mx1, Mx2, OAS1, OAS2, OAS3, OAS-like (OASL), PKR, and ISG15. Induction patterns of ISGs in bat cells also appears similar to other species when induced by synthetic IFN or poly I: C. Assessing the expression profile of the black flying fox kidney cells revealed upregulation of OAS1, PKR, and Mx1 upon treatment with IFNβ and IFNλ2 (Zhou et al., 2013). *In vitro* viral infection experiments also provided evidence for induction of bat ISGs upon infection with Pteropine ortheovirus (PRV) NB which induced Mx1, OAS1 and PKR genes (Zhou et al., 2013). Upon stimulation with poly I:C, big brown bat kidney cells were found to express the transcripts for MDA5, RIG-1, radical S-adenosyl methionine domain containing 2 (RSAD2), IRF7, OAS1, IFN-inducible protein 6 (IFIT6) and Mx1. Out of all of the bat ISGs that have been examined to date, their sequence patterns appear conserved, with the exception of ISG15, which has undergone positive selection in the black flying fox (Zhang et al., 2013a). This gene has been proven to enhance the IFN response in mice, however its role in bats is yet to be studied but could suggest a similar advantageous role.

### Novel Bat Interferon-Stimulated Genes

It has been hypothesized that in addition to the ISGs that bats share with other mammals, they possess a small amount of a special subset of ISGs that may have key roles in limiting viral replication and therefore contributing to their unique host-virus co-existence. Gene expression analyses have revealed a small number of novel ISGs that appear to be unique to bats, one of which is called ribonuclease L (RNASEL), which is not found in humans but is highly inducible in the black flying fox. RNASEL encodes a 2’5’-oligoadenylate synthetase-dependent RNase, which is a key protein in the antiviral response that degrades viral RNA in response to 2’5’-linked oligoadenylates produced by the OAS family of enzymes when stimulated by dsRNA from viruses (De La Cruz-Rivera et al., 2018). The induction of RNASEL in response to IFN in bats may give an extra layer of antiviral protection as knock-out experiments showed that black flying fox cells lacking RNASEL had an increased susceptibility to viral infection (De La Cruz-Rivera et al., 2018). Another study conducted on Jamaican fruit bats (*Artibeus jamaicensis*) also found an increased RNASEL level in the spleen following infection with Tacaribe virus, indicating that RNASEL induction in bats can be observed *in vivo* (Zhang et al., 2013a). In humans, only upstream proteins of OAS are induced by IFNs, whereas bats appear to induce both parts of the OAS/RNASEL pathway which is likely to induce a quicker effect to hinder viral replication before it can spread into further neighboring cells (De La Cruz-Rivera et al., 2018). Other possible unique ISGs that have been identified in the black flying fox via examination of their gene expression profiles include; EMC2, FILIP1, IL17RC, OTOGL, SLC10A2, and SLC4A1, but it is currently unknown whether these genes are expressed only in certain cells or certain species of bat (De La Cruz-Rivera et al., 2018). Additionally, molecular mechanisms of antiviral actions of these novel ISGs warrant future investigations.

### Interferon-Stimulated Gene Expression in Bats

It has been recognized that in IFN-stimulated cells, bat ISGs fall into two categories that share similar early induction kinetics but possess a unique late phase decline (De La Cruz-Rivera et al., 2018). These findings are significant as this decline phase is not present in human cells, therefore bat ISG levels appear to remain elevated for longer than their human counterparts. Many studied genes had higher induction levels in comparison with human cells, suggesting that bat ISGs may provide some residual antiviral protection even when IFN signaling is returned to basal levels. This proposes possible evidence for any species-specific differences in viral susceptibility (De La Cruz-Rivera et al., 2018). It is currently unclear why only certain ISGs are temporally induced in this manner and further research is required to determine whether these are unique products of IFN induction in bats. Bat ISGs that are conserved between other species, have also been found to be expressed in higher levels than their human counterparts, which again may be another unique immune feature contributing to the viral-host relationship observed in bats (De La Cruz-Rivera et al., 2018). Overall, several novel ISGs and their atypical induction has been identified in bat cells. However, homologues of atypical ISGs expressed in human cells has not yet been studied. This could prove useful in allowing researchers to design strategies to induce or exogenously activate antiviral pathways in other animals and humans that are infected with viruses (Banerjee et al., 2020).

## Future Prospects

Until recently, bats have remained one of the least extensively studied mammals but have been proven as vital components in the transmission of many emerging and re-emerging diseases. With the recent COVID-19 outbreak originating from bats, it is now more important than ever to focus time and interest into bats as viral reservoirs to gain an understanding of their immunology in hope to reduce the emergence of new viruses from bats and manage their spread in future. Most bat species studied to date appear to share a myriad of immunological features with humans and other mammals. However, studies already conducted on bat immunity suggest that even though they share many key immune features to their human counterparts, bats also possess so-called “unique” immune characteristics and functional differences in the regulation of their innate immune system. It has been hypothesized that these unique immune features may allow bats to attain a symbiotic viral-host relationship whereby despite being infected with the virus, they do not display any clinical signs of disease and still retain the ability to transmit the virus to other species. Many hypotheses as to why studied bat species possess these novel immune characteristics refer to their novel behavioral features, such as flight capability, nocturnal activities, mating, and geographical distribution (O’Shea et al., 2014). These factors may all underlie or contribute to the immune mechanisms utilized by bats to give them their novel innate immune response to viruses. Although many of these immune characteristics have been unearthed, more research into their applications is required. For example, the observation that the black flying fox expresses high baseline levels of IFNα, which may provide them with an advantageous system for controlling viral replication, contradicts the reduced levels of IFNβ also detected in the Egyptian fruit bat. Conceivably, bats may manage an inimitable balance between the levels of the two key type I IFNs, aiding in their antiviral abilities. However, further research is required to make this conclusive and to comparatively assess the constitutive or inducible expression of IFNs in all bat species, not just in the black flying fox and Egyptian fruit bats.

There are many challenges to overcome when studying bats. Despite the enticing information already gained about bats and their immunity, results have only been obtained from a limited number of studies due to lack of previous information available. There is currently a limited repertoire of cell lines from very few selected bat species available for studies, which is an issue as results can cause bias toward certain bat species and hence cannot be taken as representative of the Chiroptera order as a whole. Lack of availability of material has also led to studies where viruses have been propagated and cell lines used that have been derived from unrelated or closely related bat species. Therefore, despite finding immune factors in model species experiments, namely, in the black flying fox, it is not acknowledged that this would be the case in all other species of bat. Although many *in vitro* experiments have proved successful in proving the identification of the bat IFN system and signaling, these results are not reflective of bats in the wild, as there are many discrepancies between the behaviors and characteristics of those observed in the lab in comparison to bats in their natural habitats. For example, it is critical to recognize that in the wild, bats, and other wild animals, are often infected with a multitude of viruses, bacteria, protozoans, and helminths. This heterogeneity is often lost in lab experiments. Thereby zoonotic viruses introduced to bats in natural conditions may face very different immune states to those conduced in a laboratory. Furthermore, many experiments conducted in the laboratory have used bat cells alongside human isolates of closely related viruses due to the inability to isolate most bat-borne viruses from their original source. These investigations demonstrated that infected bats do not develop the disease, causing speculations of bats as the reservoir hosts of the virus. Isolating the original bat-borne virus from the source species is required to provide a more reliable and definitive representation (Banerjee et al., 2020). Most infection studies conducted in bats or bat cells have used human isolates and virus stocks that have been propagated from non-bat cell lines. By doing so in non-natural hosts, adaptive mutations are generated in the virus which over time cause the lab cultures to no longer represent the original viral isolate from bats. To attain more representative results from bat studies, there is a desperate desire to obtain direct viral isolates from bat hosts in the wild, which poses an obvious challenge for researchers and data collection.

One of the main limiting factors in the study of bats is their incomplete genomic annotation. The available genomes are limited to few bat species which presents a narrow comparison window, although the recent annotations of six bat genomes by the Bat1K project now shows promising potential, with plans to also annotate genomes of further bat species (Jebb et al., 2020). Application of genome-scale transcriptomics, genomics, and metabolomics in diverse bat species will highlight the true uniqueness against multiple zoonotic viruses. Despite bats proving to be important hosts of zoonotic viruses, there is still very little known about their host-virus relationships, largely because there are very few bat colonies available for experimentation and limited availability of reagents. With developed methods and expertise being developed, bat antiviral responses may be explored in much better detail, allowing us to gain an understanding of how bats interact with the virus and how they are transmitted between species (Schountz, 2014). Based on the previous work conducted on innate immunity in bats, notably their IFN response, bat immunity appears a highly promising study-model in providing useful insights into this fertile research area. Due to previous lack of material availability and the potential applications of the bat innate immune response, an exciting opportunity for research is now obtainable to explore the bat IFN response in more detail and gain a full understanding. With the field of bat immunology and virology now progressing at a faster rate than before, prospects for research into bat innate immunity and their unique host-virus relationship will aim to provide useful in determining the extent of their immune capabilities and their role as a key zoonotic host. Furthermore, via discovering the novel adaptations of bat immune systems, the current understanding of the human immune response may be redefined and possibly utilized when applied to other species in preventing pathology of disease.

## Author Contributions

MM and EC perceived the idea and wrote and revised the manuscript. All authors contributed to the article and approved the submitted version.

## Funding

Laboratory of Molecular Virology at the Lancaster University is funded by the Biotechnology and Biological Sciences Research Council (BBSRC) (BB/M008681/1 and BBS/E/I/00001852) and the British Council (172710323 and 332228521).

## Conflict of Interest

The authors declare that the research was conducted in the absence of any commercial or financial relationships that could be construed as a potential conflict of interest.

## References

[B1] Ahn M. Cui J. Irving A. T. Wang L. F. (2016). Unique Loss of the PYHIN Gene Family in Bats Amongst Mammals: Implications for Inflammasome Sensing. Sci. Rep. 6, 21722. 10.1038/srep21722 26906452 PMC4764838

[B2] Ahn M. Anderson D. E. Zhang Q. Tan C. W. Lim B. L. Luko K. (2019). Dampened NLRP3-mediated inflammation in bats and implications for a special viral reservoir host. Nat. Microbiol. 4 (5), 789–799. 10.1038/s41564-019-0371-3 30804542 PMC7096966

[B3] Akira S. Uematsu S. Takeuchi O. (2006). Pathogen recognition and innate immunity. Cell 124 (4), 783–801. 10.1016/j.cell.2006.02.015 16497588

[B4] Bagchi A. Herrup E. A. Warren H. S. Trigilio J. Shin H. S. Valentine C. (2007). MyD88-dependent and MyD88-independent pathways in synergy, priming, and tolerance between TLR agonists. J. Immunol. 178 (2), 1164–1171. 10.4049/jimmunol.178.2.1164 17202381

[B5] Baker M. Zhou P. (2015). “Chapter 14: Bat Immunology,” in Bats viruses, 327–348. 10.1002/9781118818824.ch14

[B6] Banerjee A. Falzarano D. Rapin N. Lew J. Misra V. (2019). Interferon Regulatory Factor 3-Mediated Signaling Limits Middle-East Respiratory Syndrome (MERS) Coronavirus Propagation in Cells from an Insectivorous Bat. Viruses 11 (2). 10.3390/v11020152 PMC6410008 30781790

[B7] Banerjee A. Zhang X. Yip A. Schulz K. S. Irving A. T. Bowdish D. (2020). Positive Selection of a Serine Residue in Bat IRF3 Confers Enhanced Antiviral Protection. iScience 23 (3), 100958. 10.1016/j.isci.2020.100958 32179480 PMC7075978

[B8] Barber G. N. (2011). Innate immune DNA sensing pathways: STING, AIMII and the regulation of interferon production and inflammatory responses. Curr. Opin. Immunol. 23 (1), 10–20. 10.1016/j.coi.2010.12.015 21239155 PMC3881186

[B9] Brzozka K. Finke S. Conzelmann K. K. (2006). Inhibition of interferon signaling by rabies virus phosphoprotein P: activation-dependent binding of STAT1 and STAT2. J. Virol. 80 (6), 2675–2683. 10.1128/jvi.80.6.2675-2683.2006 16501077 PMC1395475

[B10] Calisher C. H. Childs J. E. Field H. E. Holmes K. V. Schountz T. (2006). Bats: important reservoir hosts of emerging viruses. Clin. Microbiol. Rev. 19 (3), 531–545. 10.1128/CMR.00017-06 16847084 PMC1539106

[B11] Cogswell-Hawkinson A. Bowen R. James S. Gardiner D. Calisher C. H. Adams R. (2012). Tacaribe virus causes fatal infection of an ostensible reservoir host, the Jamaican fruit bat. J. Virol. 86 (10), 5791–5799. 10.1128/JVI.00201-12 22379103 PMC3347293

[B12] Cowled C. Baker M. Tachedjian M. Zhou P. Bulach D. Wang L. F. (2011a). Molecular characterisation of Toll-like receptors in the black flying fox Pteropus alecto. Dev. Comp. Immunol. 35 (1), 7–18. 10.1016/j.dci.2010.07.006 20692287 PMC7103217

[B13] Cowled C. Baker M. Zhou P. Tachedjian M. Wang L.-F. (2011b). Molecular characterisation of RIG-I-like helicases in the black flying fox, Pteropus alecto. Dev. Comp. Immunol. 36, 657–664. 10.1016/j.dci.2011.11.008 22166340 PMC7103216

[B14] De La Cruz-Rivera P. C. Kanchwala M. Liang H. Kumar A. Wang L. F. Xing C. (2018). The IFN Response in Bats Displays Distinctive IFN-Stimulated Gene Expression Kinetics with Atypical RNASEL Induction. J. Immunol. 200 (1), 209–217. 10.4049/jimmunol.1701214 29180486 PMC5736455

[B15] Dempsey A. Bowie A. G. (2015). Innate immune recognition of DNA: A recent history. Virology 479–480, 146–152. 10.1016/j.virol.2015.03.013 PMC4424081 25816762

[B16] Devaux C. Serra-Cobo J. Frutos R. Afelt A. (2018). “Chapter 8: Bats, Bat-Borne Viruses, and Environmental Changes Bats, Bat-Borne Viruses, and Environmental Changes,” in Bats, 113–132.

[B17] Hartlova A. Erttmann S. F. Raffi F. A. Schmalz A. M. Resch U. Anugula S. (2015). DNA damage primes the type I interferon system via the cytosolic DNA sensor STING to promote anti-microbial innate immunity. Immunity 42 (2), 332–343. 10.1016/j.immuni.2015.01.012 25692705

[B18] Hawkins J. A. Kaczmarek M. E. Muller M. A. Drosten C. Press W. H. Sawyer S. L. (2019). A metaanalysis of bat phylogenetics and positive selection based on genomes and transcriptomes from 18 species. Proc. Natl. Acad. Sci. U.S.A. 116 (23), 11351–11360. 10.1073/pnas.1814995116 31113885 PMC6561249

[B19] He X. Korytar T. Schatz J. Freuling C. M. Muller T. Kollner B. (2014). Anti-lyssaviral activity of interferons kappa and omega from the serotine bat, Eptesicus serotinus. J. Virol. 88 (10), 5444–5454. 10.1128/JVI.03403-13 24574413 PMC4019090

[B20] Hoebe K. Du X. Georgel P. Janssen E. Tabeta K. Kim S. O. (2003). Identification of Lps2 as a key transducer of MyD88-independent TIR signalling. Nature 424 (6950), 743–748. 10.1038/nature01889 12872135

[B21] Holland R. A. (2007). Orientation and Navigation in Bats: Known Unknowns or Unknown Unknowns? Behav. Ecol. Sociobiol. 61 (5), 653–660. 10.1007/s00265-006-0297-7

[B22] Hölzer M. Schoen A. Wulle J. Müller M. A. Drosten C. Marz M. (2019). Virus- and interferon alpha-induced transcriptomes of cells from the microbat myotis daubentonii. iScience 19, 647–661. 10.1016/j.isci.2019.08.016 31465999 PMC6718828

[B23] Isaacs A. Lindenmann J. (1987). Virus interference. I. The interferon. By A. Isaacs and J. Lindenman. J. Interferon. Res. 7 (5), 429–438. 10.1089/jir.1987.7.429 2445832

[B24] Ivashkiv L. B. Donlin L. T. (2014). Regulation of type I interferon responses. Nat. Rev. Immunol. 14 (1), 36–49. 10.1038/nri3581 24362405 PMC4084561

[B25] Iwasaki A. Medzhitov R. (2004). Toll-like receptor control of the adaptive immune responses. Nat. Immunol. 5 (10), 987–995. 10.1038/ni1112 15454922

[B26] Janeway C. A. Jr. (1989). Approaching the asymptote? Evolution and revolution in immunology. Cold Spring Harb. Symp. Quant. Biol. 54 Pt 1, 1–13. 10.1101/sqb.1989.054.01.003 2700931

[B27] Jebb D. Huang Z. Pippel M. Hughes G. M. Lavrichenko K. Devanna P. (2020). Six reference-quality genomes reveal evolution of bat adaptations. Nature 583 (7817), 578–584. 10.1038/s41586-020-2486-3 32699395 PMC8075899

[B28] Kawasaki T. Kawai T. (2014). Toll-like receptor signaling pathways. Front. Immunol. 5, 461. 10.3389/fimmu.2014.00461 25309543 PMC4174766

[B29] Kepler T. B. Sample C. Hudak K. Roach J. Haines A. Walsh A. (2010). Chiropteran types I and II interferon genes inferred from genome sequencing traces by a statistical gene-family assembler. BMC Genomics 11 (1), 444. 10.1186/1471-2164-11-444 20663124 PMC3091641

[B30] Killip M. J. Fodor E. Randall R. E. (2015). Influenza virus activation of the interferon system. Virus Res. 209, 11–22. 10.1016/j.virusres.2015.02.003 25678267 PMC4638190

[B31] Kohl C. Brinkmann A. Radonić A. Dabrowski P. W. Nitsche A. Mühldorfer K. (2020). Zwiesel bat banyangvirus, a potentially zoonotic Huaiyangshan banyangvirus (Formerly known as SFTS)-like banyangvirus in Northern bats from Germany. Sci. Rep. 10 (1), 1370. 10.1038/s41598-020-58466-w 31992832 PMC6987236

[B32] Le Page C. Genin P. Baines M. G. Hiscott J. (2000). Interferon activation and innate immunity. Rev. Immunogenet. 2 (3), 374–386. 11256746

[B33] Lei M. Dong D. (2016). Phylogenomic analyses of bat subordinal relationships based on transcriptome data. Sci. Rep. 6, 27726. 10.1038/srep27726 27291671 PMC4904216

[B34] Liu S. Cai X. Wu J. Cong Q. Chen X. Li T. (2015). Phosphorylation of innate immune adaptor proteins MAVS, STING, and TRIF induces IRF3 activation. Science 347 (6227), aaa2630. 10.1126/science.aaa2630 25636800

[B35] Majer O. Liu B. Barton G. M. (2017). Nucleic acid-sensing TLRs: trafficking and regulation. Curr. Opin. Immunol. 44, 26–33. 10.1016/j.coi.2016.10.003 27907816 PMC5446938

[B36] Mogensen T. H. (2009). Pathogen recognition and inflammatory signaling in innate immune defenses. Clin. Microbiol. Rev. 22 (2), 240–273. 10.1128/CMR.00046-08 19366914 PMC2668232

[B37] Negredo A. Palacios G. Vázquez-Morón S. González F. Dopazo H. Molero F. (2011). Discovery of an ebolavirus-like filovirus in europe. PLoS Pathog. 7 (10), e1002304. 10.1371/journal.ppat.1002304 22039362 PMC3197594

[B38] Ning S. Pagano J. S. Barber G. N. (2011). IRF7: activation, regulation, modification and function. Genes Immun. 12 (6), 399–414. 10.1038/gene.2011.21 21490621 PMC4437765

[B39] Olson M. V. (1999). When less is more: gene loss as an engine of evolutionary change. Am. J. Hum. Genet. 64 (1), 18–23. 10.1086/302219 9915938 PMC1377697

[B40] Omatsu T. Bak E. J. Ishii Y. Kyuwa S. Tohya Y. Akashi H. (2008). Induction and sequencing of Rousette bat interferon α and β genes. Vet. Immunol. Immunopathol. 124, 169–176. 18436311 10.1016/j.vetimm.2008.03.004 PMC7112530

[B41] Onoguchi K. Yoneyama M. Takemura A. Akira S. Taniguchi T. Namiki H. (2007). Viral infections activate types I and III interferon genes through a common mechanism. J. Biol. Chem. 282 (10), 7576–7581. 10.1074/jbc.M608618200 17204473

[B42] O’Neill L. A. Bowie A. G. (2007). The family of five: TIR-domain-containing adaptors in Toll-like receptor signalling. Nat. Rev. Immunol. 7 (5), 353–364. 10.1038/nri2079 17457343

[B43] O’Shea T. J. Cryan P. M. Cunningham A. A. Fooks A. R. Hayman D. T. Luis A. D. (2014). Bat flight and zoonotic viruses. Emerg. Infect. Dis. 20 (5), 741–745. 10.3201/eid2005.130539 24750692 PMC4012789

[B44] Paludan S. R. Bowie A. G. (2013). Immune sensing of DNA. Immunity 38 (5), 870–880. 10.1016/j.immuni.2013.05.004 23706668 PMC3683625

[B45] Papenfuss A. T. Baker M. L. Feng Z. P. Tachedjian M. Crameri G. Cowled C. (2012). The immune gene repertoire of an important viral reservoir, the Australian black flying fox. BMC Genomics 13, 261. 10.1186/1471-2164-13-261 22716473 PMC3436859

[B46] Pavlovich S. S. Lovett S. P. Koroleva G. Guito J. C. Arnold C. E. Nagle E. R. (2018). The Egyptian Rousette Genome Reveals Unexpected Features of Bat Antiviral Immunity. Cell 173 (5), 1098–1110 e1018. 10.1016/j.cell.2018.03.070 29706541 PMC7112298

[B47] Pippig D. A. Hellmuth J. C. Cui S. Kirchhofer A. Lammens K. Lammens A. (2009). The regulatory domain of the RIG-I family ATPase LGP2 senses double-stranded RNA. Nucleic Acids Res. 37 (6), 2014–2025. 10.1093/nar/gkp059 19208642 PMC2665237

[B48] Randall R. E. Goodbourn S. (2008). Interferons and viruses: an interplay between induction, signalling, antiviral responses and virus countermeasures. J. Gen. Virol. 89 (Pt 1), 1–47. 10.1099/vir.0.83391-0 18089727

[B49] Rocha R. Aziz S. Brook C. Carvalho W. Cooper-Bohannon R. Frick W. (2020). Bat conservation and zoonotic disease risk: a research agenda to prevent misguided persecution in the aftermath of COVID-19. Anim. Conserv. 10.1111/acv.12636

[B50] Sarkis S. Lise M. C. Darcissac E. Dabo S. Falk M. Chaulet L. (2018). Development of molecular and cellular tools to decipher the type I IFN pathway of the common vampire bat. Dev. Comp. Immunol. 81, 1–7. 10.1016/j.dci.2017.10.023 29122634

[B51] Schoggins J. W. Rice C. M. (2011). Interferon-stimulated genes and their antiviral effector functions. Curr. Opin. Virol. 1 (6), 519–525. 10.1016/j.coviro.2011.10.008 22328912 PMC3274382

[B52] Schountz T. (2014). Immunology of bats and their viruses: challenges and opportunities. Viruses 6 (12), 4880–4901. 10.3390/v6124880 25494448 PMC4276934

[B53] Sen G. C. (2001). Viruses and Interferons. Annu. Rev. Microbiol. 55 (1), 255–281. 10.1146/annurev.micro.55.1.255 11544356

[B54] Shaw A. E. Hughes J. Gu Q. Behdenna A. Singer J. B. Dennis T. (2017). Fundamental properties of the mammalian innate immune system revealed by multispecies comparison of type I interferon responses. PLoS Biol. 15 (12), e2004086. 10.1371/journal.pbio.2004086 29253856 PMC5747502

[B55] Stewart W.E II A.R.S.S.E. (1969). Persistant infection in bats and bat cell cultures with Japanese encephalitis virus. Bacterial Proc.

[B56] Thomas S. P. Suthers R. A. (1972). The Physiology and Energetics of Bat Flight. J. Exp. Biol. 57, 317–335.

[B57] Thompson M. R. Kaminski J. J. Kurt-Jones E. A. Fitzgerald K. A. (2011). Pattern recognition receptors and the innate immune response to viral infection. Viruses 3 (6), 920–940. 10.3390/v3060920 21994762 PMC3186011

[B58] Uze G. Monneron D. (2007). IL-28 and IL-29: newcomers to the interferon family. Biochimie 89 (6-7), 729–734. 10.1016/j.biochi.2007.01.008 17367910

[B59] Virtue E. R. Marsh G. A. Baker M. L. Wang L. F. (2011). Interferon production and signaling pathways are antagonized during henipavirus infection of fruit bat cell lines. PLoS One 6 (7), e22488. 10.1371/journal.pone.0022488 21811620 PMC3139658

[B60] Wang L. F. Anderson D. E. (2019). Viruses in bats and potential spillover to animals and humans. Curr. Opin. Virol. 34, 79–89. 10.1016/j.coviro.2018.12.007 30665189 PMC7102861

[B61] Wang W. Yang Y. Li L. Shi Y. (2003). Synleurin, a novel leucine-rich repeat protein that increases the intensity of pleiotropic cytokine responses. Biochem. Biophys. Res. Commun. 305 (4), 981–988. 10.1016/S0006-291X(03)00876-3 12767927

[B62] Weber F. Kochs G. Haller O. (2004). Inverse interference: how viruses fight the interferon system. Viral Immunol. 17 (4), 498–515. 10.1089/vim.2004.17.498 15671747

[B63] Wu F. Zhao S. Yu B. Chen Y. M. Wang W. Song Z. G. (2020). A new coronavirus associated with human respiratory disease in China. Nature 579 (7798), 265–269. 10.1038/s41586-020-2008-3 32015508 PMC7094943

[B64] Xia P. Wang S. Gao P. Gao G. Fan Z. (2016). DNA sensor cGAS-mediated immune recognition. Protein Cell 7 (11), 777–791. 10.1007/s13238-016-0320-3 27696330 PMC5084157

[B65] Xie J. Li Y. Shen X. Goh G. Zhu Y. Cui J. (2018). Dampened STING-Dependent Interferon Activation in Bats. Cell Host. Microbe 23 (3), 297–301 e294. 10.1016/j.chom.2018.01.006 29478775 PMC7104992

[B66] Zhang Y. B. Hu C. Y. Zhang J. Huang G. P. Wei L. H. Zhang Q. Y. (2003). Molecular cloning and characterization of crucian carp (Carassius auratus L.) interferon regulatory factor 7. Fish Shellfish Immunol. 15 (5), 453–466. 10.1016/s1050-4648(03)00025-1 14550671

[B67] Zhang G. Cowled C. Shi Z. Huang Z. Bishop-Lilly K. A. Fang X. (2013a). Comparative analysis of bat genomes provides insight into the evolution of flight and immunity. Science 339 (6118), 456–460. 10.1126/science.1230835 23258410 PMC8782153

[B68] Zhang Y. He X. Yu F. Xiang Z. Li J. Thorpe K. L. (2013b). Characteristic and functional analysis of toll-like receptors (TLRs) in the lophotrocozoan, Crassostrea gigas, reveals ancient origin of TLR-mediated innate immunity. PLoS One 8 (10), e76464. 10.1371/journal.pone.0076464 24098508 PMC3788107

[B69] Zhou P. Cowled C. Marsh G. A. Shi Z. Wang L. F. Baker M. L. (2011a). Type III IFN receptor expression and functional characterisation in the pteropid bat, Pteropus alecto. PLoS One 6 (9), e25385. 10.1371/journal.pone.0025385 21980438 PMC3181264

[B70] Zhou P. Cowled C. Todd S. Crameri G. Virtue E. R. Marsh G. A. (2011b). Type III IFNs in pteropid bats: differential expression patterns provide evidence for distinct roles in antiviral immunity. J. Immunol. 186 (5), 3138–3147. 10.4049/jimmunol.1003115 21278349 PMC3057921

[B71] Zhou P. Cowled C. Wang L. F. Baker M. L. (2013). Bat Mx1 and Oas1, but not Pkr are highly induced by bat interferon and viral infection. Dev. Comp. Immunol. 40 (3-4), 240–247. 10.1016/j.dci.2013.03.006 23541614

[B72] Zhou P. Cowled C. Mansell A. Monaghan P. Green D. Wu L. (2014). IRF7 in the Australian black flying fox, Pteropus alecto: evidence for a unique expression pattern and functional conservation. PLoS One 9 (8), e103875. 10.1371/journal.pone.0103875 25100081 PMC4123912

[B73] Zhou P. Tachedjian M. Wynne J. W. Boyd V. Cui J. Smith I. (2016). Contraction of the type I IFN locus and unusual constitutive expression of IFN-alpha in bats. Proc. Natl. Acad. Sci. U.S.A. 113 (10), 2696–2701. 10.1073/pnas.1518240113 26903655 PMC4790985

[B74] Zhou P. Yang X. L. Wang X. G. Hu B. Zhang L. Zhang W. (2020). A pneumonia outbreak associated with a new coronavirus of probable bat origin. Nature 579 (7798), 270–273. 10.1038/s41586-020-2012-7 32015507 PMC7095418

